# Salicornia: evaluating the halophytic extremophile as a food and a pharmaceutical candidate

**DOI:** 10.1007/s13205-016-0418-6

**Published:** 2016-04-18

**Authors:** Seema Patel

**Affiliations:** Bioinformatics and Medical Informatics Research Center, San Diego State University, 5500 Campanile Dr, San Diego, CA 92182 USA

**Keywords:** *Salicornia*, Halophyte, Food plant, Dietary fiber, Saponin

## Abstract

Food insecurity is a major issue in current scenario where a large section of mankind is at risk of insufficient diet. As food productivity has its limits, the prospecting of unutilized or underutilized flora as food candidates is collectively recognized as a sustainable option. In the past decade, a number of obscure plants have been identified to be rich in dietary components and deemed fit for integration into the food platter. This review discusses a candidate Salicornia, belonging to family Amaranthaceae. This halophyte has a broad geographical distribution, and phytochemical profiling has indicated its food relevance. An array of functional nutrients as fibers, polyphenols, and flavonoids have been detected in Salicornia. Though high salt, oxalate and saponin content in the plants are anti-nutrients, they can be removed to justify usage of Salicornia as a ‘sea vegetable’. Apart from culinary relevance, medicinal attributes like immunomodulatory, lipid-lowering, antiproliferative, osteoprotective, and hypoglycemic render this lesser-known marsh plant significant for phytochemical studies. This appraisal is expected to be useful towards further research and popularization of this extremophile halophyte.

## Introduction

**Fig. 1:**
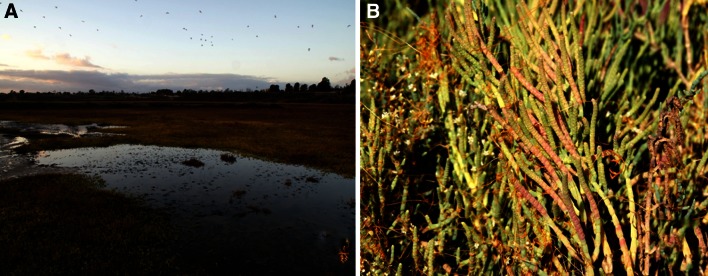
**a** *Salicornia* blanketing a marsh in Upper Newport Bay, California. **b** *Salicornia* infected by *Cuscuta*

**Fig. 2:**
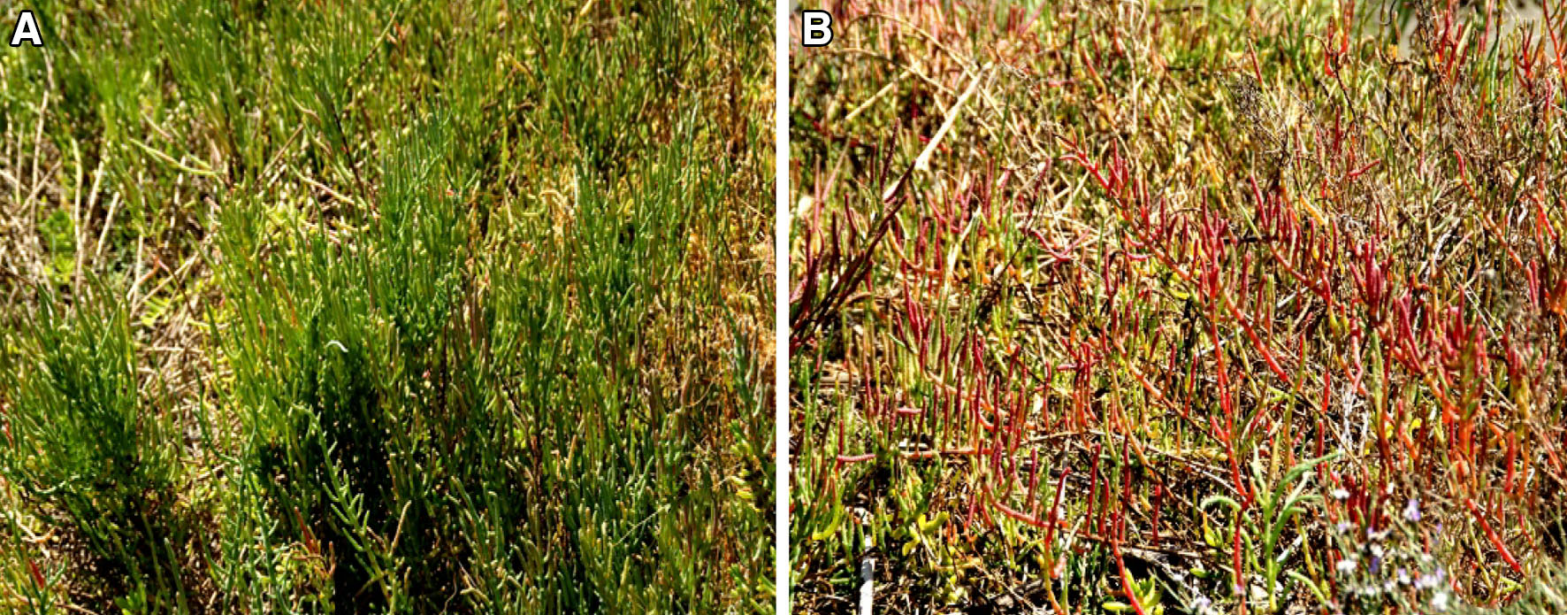
**a** *Salicornia* in spring and summer is *green* and fit for consumption, **b** *Salicornia* in autumn is *red* and *purple*, with high salt concentration, is not suitable for food purpose

Salicornia, also commonly and variably known as pickleweed, glasswort, sea beans, sea asparagus, crow’s foot greens, and samphire is a halophyte, belonging to Amaranthaceae family (Singh et al. 2014). In fact, Salicornia name has originated from the Latin word meaning ‘salt’. Studies report that some species, for example *Salicornia europaea* show tolerance towards salinity as high as 3 % NaCl (Yamamoto et al. 2009). This fleshy plant is found at the edges of wetlands, marshes, sea shores, and mudflats (Fig. 1a), actually on most alkaline flats (Smillie 2015). It has a geographical distribution spanning 4 continents such as North America, Asia, Africa and Europe. This plant has spongy stems with diminutive scale-like leaves, inconspicuous flowers and fruits. The green plant turns orange, pink to reddish in autumn, before dying in winter (Fig. 2a, b). The common Salicornia species with their botanical names, common names and geographical distribution have been presented in Table 1.

**Table 1:** Most studied species of Salicornia and their geographical distributions

No.	Botanical name	Common names	Geographical Range	References
1	*Salicornia europaea*	Common glasswort	Britain, France, Ireland	Zhang et al. (2014)
2	*Salicornia bigelovii*	Dwarf glasswort	USA, Mexico	Zhang et al. (2015)
3	*Salicornia brachiata*	Umari keerai	India	Jha et al. (2012)
4	*Salicornia virginica*	American glasswort, pickleweed	Canada, USA, Mexico	Rosso et al. (2005)
5	*Salicornia maritima*	Slender glasswort	Canada, USA, Mexico	–
6	*Salicornia ramosissima*	Purple glasswort	France, Iberia	Isca et al. (2014)
7	*Salicornia herbacea*	–	Korea	Cho et al. (2015)
8	*Salicornia persica*	–	Iran	Singh et al. (2014)

Salicornia has been historically used for both non-edible and edible purposes. Usage of the plant as a source of soda (sodium carbonate) for glass making dates back to centuries. Oriental pharmacopeia reports its medicinal uses. The efficacy of *Salicornia herbacea* against oxidative stress, inflammation, diabetes, asthma, hepatitis, cancer, gastroenteritis has been reported (Essaidi et al. 2013). Food use was not altogether new, with multiple reports of their consumption as a salt source. However, recent dearth in food availability, quest for sustainable food sources and foraging interest has pushed this genus to prominence. This plant’s aerial parts are consumed in salads or processed into pickles, beverages etc.; an interest that has taken off only in the last few years. This review explores the present status of this genus in the food arena and evaluates its scope ahead.

### Food uses

For its saltiness and crunchiness, it is used as a green salad. Even in some cultures, it is considered a delicacy. Only the green, tender parts are recommended for edibility, the reddish being too high in salinity and silica. In some communities, the shoots are processed into beverages like nuruk (a type of fermentation starter), makgeolli (a Korean rice wine), or vinegar (Song et al. 2013; Kim et al. 2013). A study found that Salicornia not only stimulates the fermenting microbe propagation but also improves the quality of vinegar (Seo et al. 2010). Apart from direct consumption, these plants have been found fitting as a source of dietary salt. *S. herbacea* powder was transformed into spherical granules, which showed potential to be used like NaCl (Shin and Lee 2013). A study found that 1.5 % of Salicornia salt as a partial substitute for NaCl can be added to frankfurters for texture improvement without any perceivable side effects (Kim et al. 2014). The positive effect of the fortification was manifested in increased cooking yield and emulsion stability (Kim et al. 2014). In another interesting study, *Salicornia bigelovii* salt was discovered to prevent hypertensive effect, normally associated with NaCl. Ameliorative effect on kidney and liver was established which correlated to lower serum creatinine level (Zhang et al. 2015). Further, superoxide dismutase (SOD) and Na(+)-K(+)-ATPase activity increased while malondialdehyde (MDA) content decreased, suggesting beneficial effect on antioxidant profile of the body (Zhang et al. 2015). The overall food value of Salicornia has got a boost from the phytochemical profiling studies that have unveiled an array of nutritive components, as discussed below.

### Phytochemicals

Salicornia plants have been screened for phytochemical profile and presence of a range of carbohydrates, proteins, oils, phenolic compounds, flavonoids, sterols, saponins, alkaloids, and tannins have been reported. Water and alcohol extraction followed by component profiling has indexed many potentially bioactive compounds. Studies have reported the presence of dietary fibers, bioactive polysaccharides, proteins, lipids, sterols, flavonoids, and minerals (Mg, Ca, Fe, K) in *S. herbacea* (Essaidi et al. 2013).

An immunomodulatory polysaccharide was isolated from *S. herbacea* (Lee et al. 2006; Im et al. 2006). Monosaccharide composition analysis of *Salicornia brachiata* fraction revealed the dominance of rhamnose, arabinose, mannose, galactose, and glucose, with meager presence of ribose and xylose (Sanandiya and Siddhanta 2014). Proteomic analysis (matrix-assisted laser desorption/ionization-time of flight (MALDI-TOF) mass spectrometry) revealed high protein contents of the seeds. Based on the detection of high disulfide linkages in the globulin proteins, it was deemed suitable for consumption, as sulfur-rich proteins are generally fit for nutrition (Jha et al. 2012). Gas chromatography mass spectrometry (GC–MS) was used to profile lipidome of *S. ramosissima* which showed esterified and free fatty acids, fatty alcohol, sterols, alkanes and aromatic acid derivatives. Among the dominant components, palmitic acid, tetracosanol and octacosanol were significant (Isca et al. 2014). Tetracosanol, the aliphatic alcohol has been identified to possess α-amylase ability, which makes it relevant in diabetes therapy (Jhong et al. 2009). Octacosanol, the high-molecular-weight aliphatic alcohol has been known to be a component of cholesterol-lowering drugs such as policosanol (Liu et al. 2015). By metabolomic analysis, *S. brachiata* was detected to be rich in sulfur amino acids and polyunsaturated fatty acids (PUFAs 55–64 %) (Mishra et al. 2015). Presence of selenium in *S. brachiata* was detected (Mishra et al. 2015). Selenium is an essential micronutrient for growth and robust antioxidant effects, deficiency of which has been documented to impair the immune system (Finley 2005). In this regard, it appears justified to evaluate dietary extraction of selenium from Salicornia. A study found *S. herbacea* seed oil to be stable to oxidation and eligible to be used in food processing (Choi et al. 2014). The oil composed of linoleic acid, oleic acid, arachidic acid, palmitic acid, tocopherol (α, γ, δ type), phenol, chlorophyll, and β carotene, was free of rancidity during a 60 day dark storage period (Choi et al. 2014). Stigmastanol, 24-ethyl-δ(22)-coprostenol and several other bioactive fatty alcohols were detected (Isca et al. 2014). In another study, a chlorogenic acid, 3-caffeoyl-4-dicaffeoylquinic acid was detected in *S. herbacea* extract (Hwang et al. 2010). Another study identified pentadecyl ferulate, stigmasterol, ergosterol, vanillic aldehyde and scopoletin in *S. herbacea* (Wang et al. 2013). Chromatography could detect β sitosterol (1), stigmasterol (2), uracil (3), and isorhamnetin-3-O-β-D-glucopyranoside in *S. herbacea* (Lee et al. 2004). Saponins were identified in butanol fraction of *S. herbacea* extract, some of which exerted antioxidant effect (Kim et al. 2012). Two new noroleanane-type triterpene saponins, Salbige A and B were isolated from the aerial parts of *S.* *herbacea* (Zhao et al. 2014). A new nortriterpene saponin, bigelovii D with antifungal potential was isolated from the hydroalcoholic extract of *S. bigelovii* (Shan et al. 2015).These studies contributed towards phytochemical composition of this genus and emphasized the significant role of non-targeted metabolomics in further component analysis.

### Antioxidant

Aqueous and methanol extracts of the enzyme-treated *S. herbacea* possessed potential antioxidant effects as observed in vitro on rat liver microsomal lipid peroxidation (Jang et al. 2007). The butanol fraction *S. herbacea* methanol extract exerted scavenging activities attributable to its active principle isorhamnetin 3-*O*-β-d-glucopyranoside. The glucopyranoside intervened with inflammatory pathways via manipulation of cytokine profile (Kim et al. 2009). In a rat model, *S. herbacea* vinegar when orally administered (at 7 g/kg water) showed free radical scavenging and SOD-like activities. Furthermore, the vinegar-administered rats showed higher glycogen accumulation in liver and muscles, higher lactate and ATP metabolism, promoting enzyme activities such as muscle creatine kinase and lactate dehydrogenase, whereas serum fatigue biomarkers such as ammonia, lactate and inorganic acid were markedly decreased (Cho et al. 2015).

### Antiproliferative

Polysaccharides from botanical sources exerting anticancer properties have been well-documented (Chang 2002; Patel and Goyal 2012). In this regard, many Salicornia polysaccharides have also shown promise, validated through in vitro and in vivo models. Crude as well as purified polysaccharides from *S. herbacea* (at 0.5–4 mg/ml) demonstrated anti-proliferation of human colon cancer HT-29 cells when incubated for 24–48 h (Ryu et al. 2009). The mechanism of the cancer cell death was attributed to cell cycle arrest at G2/M phase, followed by apoptosis. Expression of the p53 tumor suppressor gene and the cyclin-dependent kinase inhibitor 1 (CDK inhibitor p21) were observed (Ryu et al. 2009). In another study *S. herbacea* -derived chlorogenic acid, 3-caffeoyl, 4-dicaffeoylquinic acid exerted control on metastasis of human fibrosarcoma HT-1080 cell line (Hwang et al. 2010). The invasion restraint was mediated through the inhibition of activator protein-1 AP-1 and signaling pathways involving protein kinase C (PKC) delta (repressing phosphorylation of ERK, p38 MAPK, and JNK) and three MAPKs, culminating in reduced activation of matrix metalloproteinase (MMP)-9 (Hwang et al. 2010). Another study found that pentadecyl ferulate from *S. herbacea* ethyl acetate extract possesses antioxidant effect and exerts anticancer response towards human hepatocellular liver carcinoma HepG2 and human lung adenocarcinoma epithelial A549 cells, along with phytol and γ-linolenic acid (Wang et al. 2013). Saponins, Salbige A and B, isolated from *S. herbacea* exerted antiproliferative activities towards A549 cells, while a pheophorbide (a chlorophyll catabolite) inhibited both A549 and HepG2 cancer cells (Zhao et al. 2014).

### Lipid lowering

Hyperlipidemia is a major cause of morbidity and mortality across the globe. *S. herbacea* ethanol (50 %) extract exerted lipid lowering in mice model when prescribed for 10 weeks, via suppression of lipogenesis related genes [sterol regulatory element-binding protein 1 (SREBP1a), fatty acid synthase (FAS), and glycerol-3-phosphate acyltransferase (GAPT)] (Park et al. 2006). In addition, flavonoids from this plant were observed to exert adipogenic inhibition in 3T3-L1 adipocytes (Kong and Seo 2012). The isorhamnetin compound reduced adipogenic differentiation by down-regulation of peroxisome proliferator-activated receptor-γ (PPARγ), CCAAT/enhancer-binding proteins (C/EBPα), SREBP1, and the adipocyte-specific proteins. Involvement of AMP-activated protein kinase (AMPK) was also observed (Kong and Seo 2012). In addition, 3-caffeoyl, 4-dihydrocaffeoylquinic acid extracted from this species prevented lipid accumulation by blocking SREBP-1c and FAS through LKB1/SIRT1 and AMPK activation as studied in HepG2 cells (Pil Hwang et al. 2013). Another mice study on this plant revealed that it can decrease body weight gain by controlling serum leptin and manipulating lipid synthesis genes as SREBP-1c, PPARγ and FAS. Intake of high fat diet along with the plant powder at 50 g/ kg dose conferred significantly better parameters compared to only high fat diet or high fat diet plus NaCl group (Pichiah and Cha 2015).

### Antibacterial

Methanol extract of *S. herbacea* showed antibacterial activities, mediated by interference with cytochrome P450 CYP1A2, CYP3A4 and CYP2D6 enzymes (Essaidi et al. 2013). Another study explored the possibility of developing antimicrobial nanoparticles from Salicornia. *S. brachiata*-based gold nanoparticles were analyzed through a set of standard tools, which revealed its poly-dispersed, crystalline nature and 22–35 nm size (Essaidi et al. 2013). The particles decimated tested bacteria, manifested in zone of clearance on inoculated plates. Further, the nanoparticles showed synergistic activity with fluoroquinolone antibiotic ofloxacin (Ayaz Ahmed et al. 2014). Based on these exciting results, follow-up studies ought to be pursued.

### Antidiabetic

Diabetes has assumed epidemic proportions in current times, due to pollution, and processed, calorie-rich food consumption, among other causal factors (Olokoba et al. 2012). Existing panel of antidiabetic drugs provoke side effects (Haque et al. 2011; Stein et al. 2013). In this regard, safer options to control hyperglycemia are being searched. Consequently, the ameliorative effect of *S. herbacea* powder on induced-diabetic rats was studied. When administered alone or recommended with exercise (in the form of swimming), it showed higher expression of liver and muscle glucose transporters GLUT-4 and GLUT-2 (Lee et al. 2015). Higher glycogen concentration in liver and muscle also corroborated the finding, heralding possible usage of the halophyte as an antidote to control diabetes (Lee et al. 2015).

### Hepatoprotective

Liver is a vital gland for proper functionality of the body (Jarrar et al. 2001). Most analgesics induce adverse effect on liver tissues and functions (Bessone 2010). To evaluate hepatoprotective effects of a Korean herbal drink, of which Salicornia was a constituent, this study was conducted. The multi-herb potion ‘taemyeongcheong’ was administered to acetaminophen-stressed mice. At 500 mg/kg dose, the drink conferred protective effects on mice liver. Drop in the level of oxidative enzymes as alanine aminotransferase (ALT), aspartate aminotransferase (AST), alkaline phosphatase (ALP), and lactate dehydrogenase (LDH), and elevation in the level of antioxidative enzymes as catalase, superoxide dismutase, glutathione peroxidase, and glutathione was observed. Decline in expression of hepatic mRNA levels of TNF-α, IL-1β, IL-6, COX-2, and iNOS genes were observed, which can be linked to the lower degree of lesions to liver tissue (Yi et al. 2015).

### Immunomodulatory

Polysaccharides from this plant have shown evidence of eliciting immune response. In this context, some pertinent studies have been discussed below. In an in vitro study, *S. herbacea* polysaccharides induced nitric oxide (NO) production from mouse peritoneal macrophages and mouse leukaemic monocyte macrophage RAW 264.7, through the activation of nuclear factor-kappaB/Rel (NF-kappaB/Rel) (Lee et al. 2006). Consequently, the polysaccharide stimulating macrophages that express iNOS gene came forth (Lee et al. 2006). In another study on *S. herbacea*, its polysaccharide demonstrated effect on monocyte activation and differentiation into macrophage (Im et al. 2006). RAW 264.7 cells elaborated cytokines such as tumor necrosis factor (TNF)-alpha and interleukin (IL)-1 beta, and nitric oxide (NO) when incubated with the polysaccharide. Further differentiation into macrophage was determined from higher adherence development in the monocytes (Im et al. 2006). Enhanced collagen-adherence is known to improve phagocytosis (Newman and Tucci 1990). Further work by same group of researchers reaffirmed that the purified polysaccharide worked in sync with IFN-γ to induce immune effector molecules as TNF-α, IL-1 β, and NO to differentiate the monocytes into the macrophages (Im et al. 2007).

### Osteoprotective

Bone health is crucial for they make the framework of body and enable proper muscle movement. Nutrient deficiency, genetic or geriatric conditions impose bone malfunctions. Osteoporosis is a key bone disorder, for which one causal agent has been identified as higher bone adipogenesis (differentiation of stem cells into mature adipocytes) (Pino et al. 2012). In this regard, *S. herbacea* extract was observed to inhibit adipogenesis via manipulation of PPARγ, CCAAT/enhancer-binding protein (C/EBP)α and SREBP1c. Resultantly, osteogenesis improved, as evidenced in MC3T3-E1 pre-osteoblasts. Osteoblastogenesis markers as alkaline phosphatase (ALP), bone morphogenetic protein (BMP)-2, osteocalcin and collagen type I (collagen-I) lent support to bone formation induction by *S. herbacea* extract (Karadeniz et al. 2014).

### Antiseptic food additive

Sepsis, caused by the compromised integrity of membrane barrier can be fatal (Li et al. 2009), so the discovery of the antiseptic effect of Salicornia is interesting, regarding which some relevant findings have been discussed here. High mobility group box 1 protein (HMGB1), a nuclear protein elaborated by activated leucocytes, is released in excess when inflammatory tissue damage renders the membrane porous (Tang et al. 2010; Lotze and Tracey 2005; Passali et al. 2012). In this scenario, inhibitors of HMGB1 are suggested to be a potential treatment for sepsis. *S. herbacea*-derived caffeoylated quinic acids showed anti-HMGB1 activity which exerted protection towards vasculature (Tuan et al. 2015a). The purified flavanones and chromone derivatives from the plant suppressed the release of HMGB1 in mice models, barricading the animal intestine from septic shocks (Tuan et al. 2015b). Role of the phytochemicals in hyperpermeability modulation needs to be investigated further, to better utilize the halophyte in fight against septicemia. While the precise mechanism is yet to be discovered for Salicornia, other studies have attributed anti- HMGB1 activity to be due to activated cholinergic anti-inflammatory pathway (Goldstein et al. 2007).

### Cultivation

Salicornia is mulled to be the right candidate for reclamation of barren lands, salt flats, and sea shores. In short, they can be deemed for seawater agriculture. It is suggested that as global warming threatens to submerge more landmass, and freshwater is depleting, a shift to saline crop might be a viable option (Katschnig et al. 2013). Few plants can tolerate excess salt and among them few are edible. In this context, Salicornia seems to be a right candidate for cultivation (Singh et al. 2014). Regarding cultivation, different degrees of success have been observed in different parts of the world. Some of the Salicornia species are being farmed at commercial scale, for biodiesel, animal feed, and salt and oil extraction, e.g. *S. bigelovii* (Cybulska et al. 2014). This species produces oleaginous seeds which have been evaluated to be a promising feedstock for biodiesel production (Falasca et al. 2014). Introduction of Salicornia in arid lands of Saudi Arabia and Africa is being pondered and practiced (Fedoroff et al. 2010). Heavy metal removal is another possible usage of this plant. Controlled cultivation of various species procured from various habitats showed different results. *S. bigelovii* was grown in greenhouse conditions, which reflected that crop yield can vary depending on plants sourced from different habitats. Greenhouse milieu reduced biomass and fruit yield, though cultivation was successful (Bresdin et al. 2016). In addition, species-specific yield was observed, as seen with *S. ramosissima*, which produced more harvestable biomass than *S. dolichostachya* (Singh et al. 2014). Even if subsequent research dismisses Salicornia as unfit for human consumption, they might be purposed for other utilities, like biofuel harvest or livestock feeding.

### Associated risks of Salicornia diet

Though plentiful studies have established beneficial effects of Salicornia, it has its share of concerns too. It is important to be aware of possible harmful reactions before consumption. Some alarming facts have been outlined below. Accumulation of heavy metals in the vegetation is a risk to consumer health (Lei et al. 2015). Wetlands are biodiversity-rich and are critical for ecological balance, but these unique ecosystems across the world are fragile now, in the face of increased anthropogenic activities (industrial effluent release, sewage treatment etc.) (Gutzwiller and Flather 2011; Anza et al. 2014). Most wetland plants are afflicted by widespread damage including Salicornia. This succulent is easily affected by metal and oil spill stressors. A study conducted on a marsh of California showed that the species *Salicornia virginica* suffers stress from heavy metals like chromium and vanadium (Rosso et al. 2005). Another study reported *S. brachiata* to be capable of imbibing cadmium, nickel and arsenic salts (Sharma et al. 2010). Yet another study reports the suitability of different Salicornia species as biomonitors of zinc and copper, emphasizing its relevance in metal remediation from water (Smillie 2015). The possibility of using *Salicornia persica* as a biofilter in a constructed wetland for effluent water released from a recirculating mariculture system was studied in Israel (Shpigel et al. 2013). These aspects might be promising from phyto-remediation perspective, definitely not from consumption standpoint.

In addition, Chenopodiaceae (goosefoot group of plants, a part of Amaranthaceae family) members are known to contain high oxalate content, which might be harmful to consumers (Norman et al. 2013). A review has described the adverse effects of dietary oxalic acid on consumer health, by reducing calcium bioavailability, causing renal stones, stunting bone growth, preventing blood coagulation etc. (Dolan et al. 2010).

High salt content in diet is a major risk factor, especially for hypertension. Excess sodium intake is known to hamper with rennin and angiotensin homeostasis, leading to endothelial dysfunction (Drenjančević-Perić et al. 2011). Halophytes, as their name suggests are known to thrive in saline areas and imbibe salt, storing them in specialized vacuoles (Priyashree et al. 2010). Ingestion of excess salt can aggravate blood pressure. In addition, Salicornia might contain iatrogenic iodine. A case study resulted that hyperconsumption of *S. herbacea* can lead to excess iodine in body, causing hypokalemic thyrotoxic paralysis (Yun et al. 2014). Discontinuation of Salicornia intake restored the potassium level and normalized thyroid imbalance (Yun et al. 2014).

Another concern is saponin toxicity. Amaranthaceae members have been characterized to contain high quantity of saponins. These glycosides have tissue necrotic (in small intestine, liver, kidney) (Diwan et al. 2000), gut permeability alteration (Onning et al. 1996), and adjuvant potential which can provoke immune system (Rajput et al. 2007).

## Discussion

From historical usage of this halophyte for glass making, the shift towards biofuel harvest occurred (Lieth and Al Masoom 1993). Salicornia though not primarily or widely consumed; its ingestion as food and medication is, however, not altogether new. Trials and nutritional assessments on it for human edibility are novel. As outlined in above sections, Salicornia both have its pros and cons as a food candidate. Additional research might better illuminate on its relevance for consumption. In this regard, some significant areas pertaining to it have been discussed below.

Chenopodiaceae members are known to contain high amount of crude protein, sulfur and minerals (Norman et al. 2013), which goes in favor of Salicornia as an edible plant. Mucilages are plant-derived polysaccharides with myriad roles such as food thickeners, binding agents, water holding agents, emulsifier etc. (Nayak et al. 2010). In this regard, Salicornia as a source of mucilage can be assessed. Amaranthaceae family member plumed cockscomb (*Celosia argentea*) elaborates an acidic polysaccharide celosian that has been characterized to ameliorate liver injuries (Hase et al. 1997). The healing effect was linked to immunomodulating effect via tumor necrosis factor-alpha (TNF-α), interleukin-1 beta (IL-1 β) and NO production (Hase et al. 1997). Consequently, Salicornia can also be evaluated for immune-modulating polysaccharides.

Previously, the adverse effects of saponin as a dietary ingredient have been mentioned. However, medicinally, saponins are crucial with an array of their health benefits reported so far, such as cytotoxic activity (Podolak et al. 2010). The variable biological effects of saponins stem from their diverse structural configurations. Based on the hydrophobic aglycone moiety, the glycosides can be categorized as triterpenoids, steroids or glycoalkaloidss (Moses et al. 2014). Amaranthaceae member *C. argentea* seed contains triterpenoid saponins (celosin) which shows in vitro antitumor and anti-inflammatory properties (Wu et al. 2011).

Apart from the validated nutrients present in the plant, its food candidature can be assumed from its plant family Amaranthaceae. Many conventional and emerging foods are sourced from this family (Amaranthaceae), which builds trust on the food potential of Salicornia. This family is at the forefront of valuable food sources e.g. beet, spinach, amaranthus and quinoa (Délano-Frier et al. 2011). Particularly, quinoa (*Chenopodium quinoa* Willd.), a related halophyte has surged to prominence in recent times, due to its high protein, lipids, fibers, vitamins and mineral contents (Maradini Filho et al. 2015). Only a few species have been evaluated so far, other species are largely inconspicuous, though there are more than 50 species under this genus. Investigation on these species is expected to reveal myriad other biological benefits. So far, among all Saicornia species, only *S. herbacea* has been the subject of lipid lowering effect, which provides ample scope to visit the potential of other species. In recent times polysaccharides from many higher plants (Strickland 2001), mushrooms (Akramiene et al. 2007) and seaweeds (Jeong et al. 2015) have been validated as candidates as immune-therapeutics. These findings build the prospect of recruiting Salicornia as well, in immune modulation.

With due research input, this genus might be popularized for food and medicinal purposes. Pairing with compatible and complementary botanicals might improve efficacy, though cross reaction-caused adverse reactions must be monitored first.

Study on this genus is not a new area and several interesting findings have accumulated over the years. It is beyond the scope of this manuscript to furnish them all, yet it is justified to outline that this genus has been evaluated to shed light on marsh habitat loss, heavy metal accumulation and saline stress tolerance mechanisms of plants etc., Adaptation to salinity is particularly a well-pursued area, for its relevance to acclimatize vulnerable plants. A study reported of improved salt tolerance in transgenic alfalfa grass by over-expression of the *S. europaea* Na(+)/H(+) antiporter gene SeNHX1 (Zhang et al. 2014). Another study found that fresh water cultivation of Salicornia yields higher phenolic and flavonoid content over saline water cultivation (Kang et al. 2015). In addition, the fresh water-grown Salicornia demonstrated higher in vitro cytotoxic effects (Kang et al. 2015). However, it is the food and medicinal facet that needs to be intensified.

Alien plant invasions are a threat to this marsh plant, which even though at preliminary stage of nutrition research, is a validated critical component of wetland food chain. Dodder (*Cuscuta salina*), the Convolvulaceae creeper infestation on Salicornia has been observed. *C. salina* parasitizes *S. virginica*, eventually killing the latter (Pennings and Callaway 1996) (Fig. 1b).

## Conclusion

Salicornia is touted as a ‘secondary vegetable’, ‘famine food’ and ‘plant for future’. Despite multiple evidences of its health benefits it languishes as a mere marsh plant. As food insecurity looms large, such nutrition sources should not be wasted. Further, saline habitats have low agronomic relevance, so this halophyte can be cultivated to make better use of them. Further investigation in the line of the suggested area is expected to promote its popularity and provide an abundant source of nutrition in the times of ‘food insecurity’.
